# Engaging adolescents in tuberculosis and clinical trial research through drama

**DOI:** 10.1186/s13063-016-1291-7

**Published:** 2016-04-06

**Authors:** Bey-Marrié Schmidt, Amber Abrams, Michele Tameris

**Affiliations:** South African Tuberculosis Vaccine Initiative, Institute of Infectious Diseases and Molecular Medicine, University of Cape Town, Anzio Road, Observatory, Cape Town, South Africa; South African Cochrane Centre, South African Medical Research Council, P. O. Box 19070, Tygerberg, 7505 South Africa; School of Anthropology and Conservation, University of Kent, Canterbury, UK

**Keywords:** Tuberculosis, Drama, Intervention, Clinical research, Qualitative evaluation

## Abstract

**Background:**

The South African Tuberculosis Vaccine Initiative is based in Worcester where tuberculosis (TB) is endemic, and incidence rates are amongst the highest nationally. In high TB burden settings after an early childhood peak, incidence rates start to rise again in adolescents, therefore they are an important target group for tuberculosis vaccine research. In 2012, learners from a local school developed a one-off theatrical production out of an educational comic book *Carina*’*s Choice*, developed by the South African Tuberculosis Vaccine Initiative in 2010. A Wellcome Trust International Engagement grant allowed for this one-off production to be further developed, with input from university students and staff, and rolled out to schools in the Worcester area as an engagement and education intervention.

**Methods:**

Focus group feedback was used to identify key messages and to develop the play’s script. Qualitative methods were used to collect and analyse relevant data. Interviews were conducted with learner-actors, pre- and post-focus group feedback was obtained from a sample of school-going adolescents, and pre- and post-questionnaires were administered to adolescent audience members.

**Results:**

From the pre-drama focus group discussions, topics such as TB symptoms, stigma and transmission were identified as areas that needed attention. After the performances, adolescents showed improved knowledge on the identified topics and they discussed TB prevention measures. They highlighted transmission of TB during pregnancy as a further topic to be addressed in future iterations of the drama. Although stigma is a difficult phenomenon to interpret, post-drama participants understood that TB transmission could occur in all individuals. Learner-actors agreed with focus group participants that the play could impact the wider community if it were rolled out. Feedback from the South African Tuberculosis Vaccine Initiative staff verified that recruitment for an upcoming trial was facilitated by the preparedness that the play provided in recruitment areas. The study showed that before and after evaluations provide data on the usefulness of the play as an education tool.

**Conclusions:**

Theatre, presented and motivated by adolescent peers, can raise awareness of TB, and assist clinical trial preparedness and further engagement between trial staff and their trial community.

## Background

Adolescents are one of the primary target populations for new tuberculosis (TB) vaccination strategies in high TB burden settings because incidence rates start to rise again in adolescents (after the established early childhood peak). The increased incidence in late adolescence makes this age group an important target for TB vaccine and related social science research [1]. Efforts to engage with adolescents to ensure that they are fully informed about TB, vaccine trials and their rights and responsibilities with respect to clinical research are imperative. Valuable data becomes available when such efforts are combined with assessments of their effectiveness, not only in terms of education and awareness, but also in terms of overall knowledge, attitudes and perceptions about the disease in question. Although interactive methods such as role playing, video games and group work are increasingly being used in health awareness raising interventions, knowledge about their effectiveness is still limited [2].

Researchers at the South African Tuberculosis Vaccine Initiative (SATVI) have conducted two TB vaccine trials in adolescents in a semi-rural, high TB burden community in the Western Cape, South Africa, since 2005 and are currently conducting a TB vaccine trial with 990 high school learners. By way of continuous engagement with people in the immediate areas surrounding their site, SATVI ensures that potential trial participants are prepared for recruitment processes and that they are educated about TB and on their rights and responsibilities in clinical trials.

A community engagement project funded by a Stop TB grant assisted this aim in the form of a comic, *Carina*’*s Choice*, which was developed by SATVI with input from staff and the Community Advisory Board in 2010. *Carina*’*s Choice* is a story of a young mother justifying her decision to enrol her infant into a TB vaccine trial to her family and friends. Upon its dissemination, the comic received positive feedback, including the independent decision by a local arts-focused school, Worcester Secondary School (WSS), to dramatise the comic using high school pupils as performers, stage managers, and light and sound engineers. The positive reception of the single performance of this dramatisation led SATVI researchers to explore funding opportunities to support further development of the drama, and its wider roll-out. With the awarding of a Wellcome Trust International Engagement Grant, the drama’s further development and subsequent assessments of its impact were made possible. This paper describes the process for developing *Carina*’*s Choice* into a play and it reports on the play’s impact as a method of engagement for TB and clinical trials.

## Methods

The most recent dramatisation of the comic, *Carina*’*s Choice*, by high school learners involved collaboration with the University of Cape Town (UCT) Drama School and WSS. The *Carina*’*s Choice* roadshow took place in the third quarter of 2013, with 12 performances at eight high schools to a total audience of about 7500 adolescents. The performers, WSS drama pupils, were selected by their teacher to participate in the drama production. A production team was formed with representatives from WSS, SATVI and UCT Drama School to be responsible for script development, skills development, production of the play including video-recording, and project management. Senior UCT drama students, supervised by a faculty member of the UCT Drama School, provided guidance to school pupils through the various stages of drama production. The language used in the play was a colloquial, slang Afrikaans, including English words and phrases. The play consisted of age- and area-appropriate humour, and song, rap and dance elements. The key messages for the drama production were chosen using the *Carina’s Choice* comic and then validated against pre- and post-focus group input. The planned key messages on initialising the project, and prior to any community engagement, are presented in Table 1.

**Table 1:** Initial key messages of the drama production

TB symptoms and actions to take when symptoms persist
TB can be treated
There is a need for a new, more effective TB vaccine
SATVI is doing clinical research to test new vaccines
Strong emphasis on safety and ethical conduct because new TB vaccines are tested in people
Participants have rights in clinical research

The analysis in this paper relies on qualitative methods. Feedback workshops were conducted with pupils who acted in the play (student-actors). Pre- and post-focus group discussions took place with a sample of learners, while pre- and post-knowledge assessment questionnaires were administered to all learners attending the performance.

Pre- and post-drama focus groups (ranging from 8 to 15 members), a central tool in this project’s effort for continuous evaluation, were conducted by a social scientist and translator at the SATVI office. Focus group participants were drawn from three schools in the Worcester area, representing the linguistic diversity of the region (English, Afrikaans and isiXhosa). The schools were chosen based on their representing all three languages spoken in the area as well as drawing children from both higher income and middle to low income backgrounds. Participants were selected by a random selection of names from a list of adolescents who had previously expressed an interest in TB vaccine research activities, using a random number generating online tool. Each school group met separately once before and once after the play. Focus group questions were based on the production’s main themes, with the aim of determining topics of local importance related to TB and to explore TB knowledge, attitudes and perceptions amongst adolescents in Worcester.

The study protocol was approved by the UCT Human Research Ethics Committee and permission to conduct the project in the local high schools was obtained from the Department of Education. Written informed consent and assent for focus group participation were obtained by SATVI staff members in the adolescent’s home language or language of choice along with permission to audio-record the group sessions. Focus groups met at the SATVI project office after school hours. The groups were semi-structured with participatory exercises (including pile-sorting [3] and a graffiti wall – see [4] for an explanation of the graffiti wall), and a pre-developed script allowing for adaptation of content and duration according to the responses received. Focus groups were audio-recorded for transcription later.

Pre-intervention focus groups were convened with the intention of identifying gaps in current knowledge and understanding, to inform the content of the script. Post-intervention focus groups were used to identify where gaps in information might still exist in the script, to evaluate the learners’ responses to the play and to determine where future engagement and information dissemination might be usefully focused. Written informed consent of those who participated in the pre- and post-intervention focus groups was obtained by recruiters from SATVI in the language that students preferred.

The production toured eight high schools in the Cape Winelands East district: six in Worcester, and one each in Rawsonville and Robertson, chosen for proximity to SATVI and therefore, logistic ease, as well as being the planned recruitment schools of an adolescent TB vaccine trial. In addition, after the production had toured at eight schools, the learner-actors forming the production joined in informal and formal discussions about their experiences on the project. Parents of learner-actors were asked to give consent before they participated in the production. Assent was obtained from leaner-actors before focus group discussions and then, at the closing of these discussions, verbal confirmation was obtained for their inputs to be used for research purposes. The transcripts from the formal discussions also formed part of the evaluation process, as did feedback from SATVI clinical staff in the form of informal conversations and emails.

Adolescent audience members completed a pre- and post-drama questionnaire to evaluate their knowledge of TB and clinical research, drawn from validated survey questionnaires [5–8]. Parents and guardians were informed of the upcoming production and knowledge assessments through a take-home letter and given the opportunity to return a slip to opt out of their child’s participation in this survey – no parent took this opportunity. All surveys remained anonymous.

### Data collection

All interviews and focus group discussions were audio-recorded, translated, transcribed and data coded. Written records created during the sessions were retained as hard copies. Photographs were taken of images created using participatory methods during the focus groups and stored digitally. Field notes, as the responsibility of individual researchers, were password protected. Additionally, knowledge survey questionnaires were anonymous with no identifiers other than basic demographics of age, gender and school grade. These were collected by the school approximately one week after the performance and captured on an access-restricted Microsoft Access database.

### Data analysis

Transcripts from focus group discussions and hard-copy documents from the “graffiti wall” and pile-sorting activities in focus groups were reviewed for emerging themes and coded inductively. A code list was generated to identify the emergent codes and sub-codes for analysis. Coding was done by two independent reviewers (AA and BS).

## Results

### Pre-drama focus groups

The scriptwriters were given feedback about the data that resulted from analysis of the pre-play focus group discussions. Additional messages were then added to the script (Table 2) and to a pre-specified list of key messages generated prior to engagement from previous research in the area [9].

**Table 2:** Topics for clarification in the production as per pre-drama focus group interviews

Differences between TB and HIV, and their association
Cause of TB
Different types of TB
Personal protection against community TB
Information dissemination and education about TB
TB reactivation and TB reinfection
Defining stigma and addressing it
Transmission of TB
Symptoms of TB, aside from coughing
The absence of TB symptoms

From the pre-drama focus groups, it was evident that coughing was a known symptom of TB. This information was incorporated in the redevelopment of the script, so that the play developed with students’ input. For example, the rewrite of the script included a focus on what to do when someone coughed. The first song of the play states “*maak daai vensters oop*; *hoes in jou elmboog*,” which is an instruction on coughing into the elbow and opening windows to increase air circulation.

Only a few adolescents could name more than one symptom of TB, but none were able to name all of the symptoms. Thus, the production team ensured that the play worked on raising awareness of other symptoms of TB, including fatigue and weight loss.

Pre-drama focus group interviews also revealed that local knowledge on transmission of TB was limited. Questions like “You can only transfer it through your spit, right?” juxtaposed with “You can get it from sex” or “What about sharing cigarettes?”, showed that the transmission of TB, as well as how TB differed from HIV needed to be clarified in the play. Focus group participants confused the transmission vectors of TB and HIV, thinking that TB was transmitted sexually, as was HIV. This conflation of the two diseases was clarified to learner-actors during production workshops, and to the audience in the play. In fact, a specific rap song explaining the difference between TB and HIV was written by the learner-actors and performed in the play (see Table 3).

**Table 3:** Rap song explaining the difference between TB and HIV

Now with a lack of sanitation and poor ventilation
With the hubbly going round and round
You can get the V
That’s TB, not H-I-V.
But while we’re on the subject, always use a rubber
Cos unprotected sex could make you a mother (or father!)
So guard yourself against HIV
Take part in the trials with SATVI
And help us with the fight against TB.

When focus group participants were asked directly about TB-related stigma, many responded that they either did not understand the term “stigma” or that they did not think that it existed. However, proceeding discussions and comments, presented in Table 4, revealed that TB was in fact highly stigmatised and there were misconceptions about those with TB disease.

**Table 4:** Comments by participants during pre-drama focus group discussions

What must people do when they have TB and when people are talking bad things about TB? Some people are saying bad things about them.
Some people keep it a secret when they are infected with TB.
Because they don’t know if you have TB, so they talk to you and they touch you. When they find out that you have TB, they don’t want you anymore.
Because they feel … how can I say this now … they feel that they are not equal to others because they have this virus.
But sometimes you get people who are like, don’t touch me, you have TB.
They are like avoiding you, and don’t touch me.
Or they say **, don’t speak with them.
Don’t go to that girl, she’s dirty.
I think it’s the ** type of illness that they’re hiding because of what they heard. So that is why it’s making it difficult for people to take their medicine.
Ja, that’s very important. Because you were saying that sometimes people don’t like the people with TB. They say go away, and they don’t want to eat with you.

Recurring questions around the safety of sharing, for example sharing a drink or cigarette, with someone who has TB were reminiscent of fears associated with the early days of HIV. Associations between TB and HIV may cause speculation of HIV amongst those who have TB. A high TB–HIV co-infection rate may lead to confusions in biomedical processes of TB and HIV, and also in everyday language. For example, “I might be a carrier” was mentioned by one of the participants suspecting TB, since “carrier” in biomedicine is used to refer to individuals with HIV infection or other diseases. Another quote again brings to light that HIV stigma is displayed in the context of TB: “But people are ashamed because people outside are going to say, oh, you have HIV and AIDS. So people are ashamed of that, so they will not go to clinic [when they suspect TB].” Nonetheless, TB stigma was also observed outside the realm of HIV terminology, where reference was made to “farm people” (people working on a farm) as most likely having TB.

Often in the context of stigma, blame for spreading TB and blame for destabilising family income is present [10]. Participants expressed that those with TB had to be concerned about transmitting it to other family members and there was a clear acknowledgement that the household income was affected, this being attributed to the infected family member. Hence, an interesting notion of “running away from TB” emerged from the focus group discussions. In the presence of stigmatisation and blame, participants described that many people with TB would not accept their diagnosis or they would not adhere to medication regimens or even collect their medications. Others would hide their positive TB diagnosis from the public (that is, by “hiding away their medicine under their beds”).

A primary aim of *Carina*’*s Choice* was to raise awareness within the Worcester community that patients, as well as clinical trial participants, have rights. From the pre-drama focus group discussions, it became evident that participants were not vulnerable, and in fact, knew “that they had rights to confidentiality”. Adolescent participants set the pace for discussions and determined the method of engagement. For example, one participant with agreement from peers asked: “Can we get to the point? I don’t literally mean to the point; I mean can we get to the next question.” Another participant, when she felt everyone had enough of a chance to answer a discussion question would exclaim, “OK, next question!” The discussion also confirmed that misconceptions about vaccines and the role of SATVI existed. Participants thought that “SATVI is vaccinating people not to get TB” and that vaccinations from SATVI “stop you from getting TB, so we can talk to someone who has TB, but we can’t get TB”. Clearing up such misconceptions was already listed as a priority of the play.

### Post-drama focus groups

In the pre-drama focus group discussions, participants only named coughing as a symptom of TB. The discussions revealed that the spread of TB is visualised as coughing and that stigma is practised against a coughing suspect. In fact, coughing was a prominent symptom of TB mentioned by focus group participants. However, participants could list other symptoms such as fatigue, night sweats and weight loss after attending the play. Even though participants conflated TB and HIV in the initial discussions, they could differentiate the spread of TB and HIV in the post-drama discussions. For example, participants could explain that “HIV is something you get when you have sex, but TB is in the air.”

The questions posed in the pre-drama focus groups relating to transmission of TB were raised again, and led to a discussion about the ways in which adolescents could protect themselves against TB, through opening windows and seeking diagnosis and treatment early. Some participants could also make links between the symptoms of TB and progression of the disease: “If you feel that you are coughing a lot and you are feeling tired, go to the hospital to be sure … get it treated.” Focus group participants said that they remembered most of the information they learned from the play through the songs that were performed. For example, one participant remembered to be cautious of coughing from the lyrics of a song “*Hoes en koes*”, presented in the Appendix, which was performed during the play.

Although stigma is not an easy phenomenon to interpret, post-drama focus group participants revealed that they learned that TB transmission is possible to all individuals regardless of socio-economic characteristics. Participants recognised that there was a change in community attitudes towards TB-diseased individuals, but it was not clear whether this change could be attributed to the play. For example, one participant explained this change, “Ja, at first, but now they are accepting the fact that they have TB. So they are starting to treat it, so it’s not much of a problem,” and another said: “But now everyone knows if you’re having TB, it can happen to anyone. So no matter in which place you are or where you’re from, they know that you can get TB.”

Post-drama focus group participants, some of whom were different individuals from those interviewed before the play, showed the same confidence in terms of their rights and participation in research. Although one participant expressed that she was tired of being asked questions about TB, another intervened, with support from others, that not everyone felt the same way. Participants went on to give their opinions about the play, “I liked it because the play advised people” and “I was not expecting the play to be in that way. They have made it in a sort of interesting way whereby they try to make sure that everyone wants to watch the play … because then you learn stuff.”

### Post-drama interviews

In addition to focus group discussions and identification of themes from anecdotal materials by participants, learner-actors took part in a feedback workshop about their experiences in the play. Both learner-actors and focus group participants agreed that the play had the potential to impact the wider community if it were rolled out, “Because most of the people out there in the communities … so if they could see the play and understand the play, I don’t think that everyone else would be afraid to go to the clinic to test if they have TB.”

The feedback that emerged from the pre-drama focus groups contributed to the learning of learner-actors. Learner-actor feedback, after the plays had run, reflected on gaps in their knowledge before their involvement in the drama, revealing strikingly similar gaps as those identified in pre-drama focus groups. Learner-actors also shared that, besides improved knowledge of TB, they learned new theories and applications of theatre from the UCT student-actors and that health awareness can be communicated through drama. Table 5 lists the responses of learner-actors in relation to what they learned from their involvement in *Carina*’*s Choice*.

**Table 5:** Comments by learner-actors during post-drama interviews

But the students from UCT taught us about image theatre – things that we can use in our drama practical.
Now I don’t look at a production in the same way when I do my practical.
It made me more reliable because I had to look after the props and made sure that everything was okay.
I also learnt more about TB. You just hear about TB, but you don’t really know how you get TB and how you can prevent it.
Yes, I’ve also learnt that you can teach other people new things by showing it to them.
And drama gives you another view of the world. You will understand it better when you see it.
I think the songs will stay with the people for a long time, and they have learnt something from it.
Through this SATVI project I built up my self-confidence again.

Feedback from SATVI staff revealed that the play assisted recruitment because it sensitised the population to the TB epidemic and TB vaccine research. School staff were receptive to further contact with SATVI and they arranged time slots for SATVI staff to recruit interested trial volunteers.

## Discussion

As a method of engagement, the play worked. *Carina*’*s Choice* increased awareness of TB and SATVI’s research activities amongst school-attending adolescents in the Worcester area. The continuous feedback process that the research team attempted to foster with the drama development team allowed for the inclusion of topics of salience (for focus groups members) in the play’s script. As is evident from post-drama discussions and interviews, learner-actors and audience members remembered songs from the play, and learner-actors became references for TB questions within their own (self-identified) communities. Furthermore, the collaboration between UCT Drama School and WSS was successful and both parties expressed great enthusiasm for future projects as encouraged by the audience.

Through the larger process of engagement that this collaboration, driven by the clinical trial site, is trying to foster, striking information emerges about the ways in which the young people involved in our focus groups understand TB. Such information is a powerful tool in understanding the social dimensions of TB in an area with extremely high rates. Our focus group data reveal an association of TB with HIV; this link creates new forms of stigma. South Africa’s specific history in acknowledging HIV and generations of death certificates claiming deaths to be a result of TB while the presence of HIV was ignored or at least not formally documented have added to an already stigmatised ailment [11]. Confusion about TB and HIV may additionally stem from the fact that the two diseases are taught together in Life Skills Orientation classes.

The original video-recording of the production as well as versions with English and Xhosa subtitles will be made available to SATVI’s collaborators in South Africa, and within the Africa-wide TB Vaccine Sites Network (TBVACSIN; coordinated from within SATVI). Video copies will also be distributed to the Department of Health as an educational tool for use in the Life Orientation programme. The video-recording may be adapted for new environments, following input from collaborators from these sites.

### Limitations

This project aimed to engage school learners around TB and clinical trial participation in a TB trial where the focus of engagement was the development of the script for the drama and the production of the performances. This paper describes the efforts that contributed to the development of the play, as part of the ongoing and preliminary analysis built into the engagement project. The majority of the project’s budget was dedicated to the development of the play, and thus, the processual or continuous evaluation methods described in this paper were purposefully not the focus of the grant. While this project aimed to fulfil a continuous method of analysis, budgetary limitations did not allow for a social science researcher to remain engaged and attend all proceedings of the workshops aimed at developing the production. It may be argued that convenience sampling of focus group participants statistically reduced the generalisability of the information shared during these sessions, but qualitative methods adhere to a principle that if a theme arises, it is pertinent, regardless of its frequency as a concern.

In addition, budgetary limitations meant that focus group numbers and meetings had to be limited. As such, a question raised at one pre-play focus group regarding transmission of TB during pregnancy was not highlighted as an important salient topic, but in post-play focus groups it arose twice, making us flag it as a potentially important future topic. That it arose initially as a concern not high on our priority list of tasks to attend to, but then again in post-play focus groups, shows both the value of our continual assessment and review, and of incorporating more focus group engagement if future iterations of this type of engagement are going to take place. Ethnographic, long-term and action-oriented research throughout this process could provide valuable input on the experience of the learner-actors to support educational research that attributes benefits of the skill sets acquired through the study of drama for empowering health-related decision-making.

## Conclusion

The play worked as a method of engagement. As the assessment process and continuous engagement are central to the method we have developed, we encourage assessments similar to that used in the pilot phase, when rolled out to other environments, and we will make our questionnaires available for this purpose. We suggest that this project may assist in creating a valuable engagement tool for other trial communities, if used in conjunction with context-specific assessment and continuous engagement for feedback on the intervention.

## Appendix

Wie het vir julle gesé, Om in TB se pad te lé, (×2)

TB maak jou lam,

TB maak jou bang,

TB van n snies,

Laat gewig verlies, (×2)

Wie het vir julle gesé, Om in TB se pad te lé, (×2)

Haal asem my longe asem, Haal asem my longe asem,

Maak daai vensters oop, (hoes)

In jou elmboog, (hoes)

Wie het vir julle gesé, Om in TB se pad te lé, (×2 Wie het; Wie Het/TB; TB)

…Hier kom daai koors,

Hy kom om die hoek,

Jy moet lekka koes, (want)

Hy gaan jou verwoes.

## Abbreviations

AIDS: acquired immune deficiency syndrome
HIV: human immunodeficiency virus
SATVI: South African Tuberculosis Vaccine Initiative
TB: tuberculosis
TBVACSIN: Tuberculosis Vaccine Sites Network
UCT: University of Cape Town
WSS: Worcester Secondary School
